# Camphor Poisoning

**Published:** 1876-08

**Authors:** 


Camphor Poisoning.—The *Druggists' Circular* contains the following: "A correspondent of the *London Pharmaceutical Journal* relates a remarkable case of poisoning, the leading features of which are as follows, and they will serve to strengthen the suggestion of the *Druggists' Circular* that especial care should be exercised in the sale of camphor: "While some camphor was being weighed out a lad of thirteen picked up two small pieces and took them away with him for the purpose of floating and burning them. Soon afterward he began nibbling the camphor, and as it afterward appeared, liking the taste of it, continued to do so until he had eaten the two pieces. This was about four o'clock in the afternoon. Four hours afterward the child was with his brother in the dispensary looking on, and was observed to do something which elicited the remark, "Are you dreaming?" No reply was given by the child, and it was noticed that something was wrong with him; his eyes were fixed in a stare, and he stood motionless and unconscious. His brother took him up to carry him in an adjoining room, where his father was, when he immediately became convulsed and perfectly rigid, with his head and legs bent back, so that he could only be placed on his side on the floor. The convulsions increased until the flesh from the head to the shoulder became purple, and the pulse decreased rapidly until it could not be felt. The body then lost its rigidity and was apparently lifeless; but in about ten seconds the pulse could again be felt, the convulsions returned, and the child foamed at the mouth. Applications of cold water brought him round in about four minutes; violent vomiting then ensued; the child was for a time hysterical, but within an hour from the first attack he was so far recovered that he could be put to bed. The child afterward described the pieces of camphor which he ate as being each half the size of his thumb, and the assistant who noticed him take out one piece thought it must have been about sixty grains in weight. The effects produced in this case were more severe than in any of those previously reported, and there was the further difference, that unconsciousness preceded the convulsions, while in the other cases it followed them."

